# A individually randomized controlled trial comparing Healer-led vs. clinician- led training to improve personal protective equipment use among traditional healers in South Africa

**DOI:** 10.1371/journal.pgph.0002945

**Published:** 2024-02-23

**Authors:** Carolyn M. Audet, Tshegofatso Seabi, Taofik Oyekunle, Jennifer Hove, Ryan G. Wagner

**Affiliations:** 1 Vanderbilt Institute for Global Health, Vanderbilt University, Nashville, TN, United States of America; 2 MRC/Wits Agincourt Research Unit, School of Public Health, Faculty of Health Sciences, University of Witwatersrand, Johannesburg, South Africa; Corporacion Universitaria Remington, COLOMBIA

## Abstract

Like allopathic healthcare workers, healers are also exposed to patients’ blood and body fluids. A widespread practice is the traditional “injection,” in which the healer performs subcutaneous incisions to rub herbs directly into the bloodied skin, resulting in 1,500 blood exposures over their lifetime. We tested the impact of healer-led PPE training, staffed by trained traditional healers who reported using PPE during each risky clinical encounter vs. healthcare worker (HCW)-led PPE training sessions. We randomized 136 healers into one of the two study arms (67 in the healer-led group, 69 in the HCW-led group) and assessed the impact of trainer on PPE skills and use over a six-month period. All healers received one in-person day of didactic and practical training followed by three sessions at the healers’ home. Participants were largely female (80%), averaged 51 years old, and practiced as a healer for an average of 17 years. Almost 44% either disclosed themselves as HIV+ or received a positive HIV test result at study initiation. Healers in the HCW arm showed equivalent PPE scores as those trained by traditional healers at baseline and at seven months. Healers in both arms self-reported high levels of glove use during”injections,” with no statistical difference of use by study arm. When we assessed actual gloves and razor blades disposed of each month, a similar trend emerged. No one seroconverted during the study period. The need for PPE support among traditional healers cannot be ignored. Traditional healers can be trained to effectively disseminate PPE knowledge and skills to other traditional healers. With an estimated 200,000 traditional healers in South Africa, it is imperative that all of them have access to PPE training and supplies to prevent HIV, HCV, or HBV infections.

**Trial registration:** ClinicalTrials.gov, NCT04440813. Registered 17 June 2020, https://clinicaltrials.gov/ct2/show/NCT04440813.

## Introduction

There are more than 2 million traditional healers in sub-Saharan Africa (SSA), 200,000 of whom work in South Africa [1–5]. Many community members first seek health care services from a healer to prevent and cure ailments and receive dancing/drumming treatments and/or herbal remedies (“injected”/incised into the skin, ingested, and baths) [6]. While substantial research efforts have been expended on understanding the positive [7–16] and negative [3, 17, 18] impact that healers can have on the health of their patients, occupational hazards associated with traditional healer practices in SSA have attracted scant attention to date [18].

Like allopathic health care workers (HCWs), healers are also exposed to patients’ blood and body fluids. A widespread practice is the traditional “injection,” (described as such by traditional healers but perhaps more accurately called ‘incisions’) in which the healer performs dozens of subcutaneous incisions in order to rub herbs directly into the bloodied skin [6, 19–21]. In South Africa, 98% of healers report conducting “injections.” resulting in an estimated 1,500 blood exposures over the course of their lifetime [22]. Healers who perform these “injections” and report exposure to patient blood have higher risk of HIV infection than healers who do not [6]. Use of personal protective equipment (PPE) by traditional healers is inconsistent, due in part to lack of access and training on appropriate use and disposal [23]. Additionally, cultural and traditional practices-traditional healing practices are deeply rooted in cultural and traditional beliefs, which influence the acceptance and adoption of modern infection control measures like PPE [23]. Some healers may prioritize their traditional methods and may not perceive PPE as necessary or compatible with their healing rituals. Despite pressure to eliminate the practice of “injections,” these treatments have been used for centuries and are often the preferred and expected treatment delivery method among patients [24, 25].

Training by expert clinicians in the donning, doffing, and disposal of PPE is known to have a positive impact on correct PPE use among health care providers [26–32]. These in-person training programs are essential to reduce the risk of infection from blood-borne pathogens among providers. Recently, peer-led training to improve clinical skills and service delivery have shown equal or greater impact than traditional “expert-led” training programs [33, 34]. Peer-led training can increase intrinsic motivation, alleviate health care provider teaching burden, and provide accessible role models for learners [35]. We tested the impact of a healer-led training model (a peer model) vs. training led by expert HCWs to assess the effect of the trainer on donning, doffing and disposal of PPE as well as overall use of PPE 7-months post-training among traditional healers living in Bushbuckridge, South Africa.

## Materials and methods

### Study design and participants

#### Study design and settings

We conducted a randomized controlled trial with 136 participants (67 in healer-led group, 69 in the HCW-led group) to assess the impact of two implementation strategies on PPE use among traditional healers. The study was conducted in the rural Bushbuckridge sub-district of Mpumalanga province, South Africa. The Medical Research Council/Wits Agincourt Research Unit oversees the maintenance and operation of the Agincourt Health and Demographic Surveillance Site (HDSS) located in the sub-district. Roughly 500km northeast of Johannesburg, the unit has been engaged in population-based health- and socio-demographic research since 1992. Strong ties with the local community ensure the continual functioning and sustainability of the research. Long-standing community links and continuing presence make Agincourt a uniquely well-suited site (due to existing infrastructure, research support, and proven track record) for testing this intervention.

#### Study population

*Inclusion and exclusion criteria*. Traditional healers ≥ 18 years of age, who were currently practicing in the Bushbuckridge area, and conduct traditional “injections” were invited to participate in the study. Those who were planning to move out of the study area in the subsequent seven months were excluded from study participation.

#### Participant involvement

Traditional healers were involved in the design and conduct of this research. The focus on the research question (HIV prevention) was inspired by healers concerns about their own health (determined by >20 interviews and an HIV prevalence survey). The trial was in partnership with three traditional healers who assisted in the development of the PPE training program, identified the need for home-based individual training, and provided training for the healers randomized to peer education. Healers have been informed about the results through several community meetings. A study newsletter suitable for a low-literacy audience is being developed and will be disseminated across the region.

#### Randomization and recruitment

Healers in the region are registered with one of the local healer organizations or are otherwise known to the leaders in the region. We partnered with the Kukula Healers Organization to identify all active healers in the region. Kukula provided a list of all eligible healers within their organization practicing within the area. Recruitment began on August 23^rd^, 2021, and ended on September 30^th^, 2021. Healers were randomly assigned to the control or intervention group using Stata. The healers were contacted by study staff via phone calls and/or text messages, inviting them to participate in the initial training session and study. Healers were given several options for the initial training session to facilitate availability. Healers who participated in the initial session were provided bus or taxi fare to cover transportation costs to and from the training venue.

#### Sample size

From preliminary data collected from healers in 2019, the median proportion of glove use during “injections” was 0.44, mean 0.39, and standard deviation (SD) 0.39. Our sample size was calculated based on a two-sample t-test and incorporated these preliminary data. Assuming a standard deviation of 0.39 in both arms, we anticipated needing 61 healers per arm (a total of 122 healers) to have 80% power to detect (when alpha = 0.05) a difference of 0.2 in the proportion of glove use between HCWs and healer teams (e.g., 0.4 vs. 0.2) (or equivalently a standardized mean difference of 0.51).

#### Study intervention

All healers received in person training based on the Centers for Disease Control and Prevention (CDC) PPE short course for healthcare personnel [36] combined with information from the World Health Organization and the Occupational Safety and Health Administration (OSHA) in August and September of 2021 [37]. The one-day (approximately 6 hour) training session was divided into didactic presentations, question and answer sessions, PPE demonstrations, and practice sessions of PPE donning, doffing and disposal. The course material covered the following elements: epidemiology, symptoms, and transmission of bloodborne pathogens; universal precautions for preventing transmission of blood borne pathogens; work practice controls; types, uses, removal, handling, decontamination, and disposal of PPE; management of regulated waste; sharps safety; and post-exposure evaluation, including linkage to care, etc. [38]. In addition to the initial training, healers received monthly visits from study team personnel to distribute new PPE and dispose of used PPE. The only difference between arms were the study team personnel leading the sessions: in the intervention arm sessions were led by three trained traditional healers, while the control arm sessions were led by three health care providers.

#### Outcome assessment

We collected used sharps and PPE from healers each month. Healers were given containers to place all used gloves and razors/sharps to both ensure healer safety and allow us to count materials used. We used the number of glove pairs as the numerator (number of times healers used gloves during procedures) and the number of razor blades as the denominator (number of “injections” given). In addition to the collection of PPE and sharps, healers self-reported their PPE use every time they conduct an “injection”. We calculated the proportion of glove use as the number of times gloves were used divided by the number of “injections” conducted each month.

#### Data analysis

Descriptive statistics for baseline characteristics were compared between the Healer- and HCW- trained traditional healers using frequencies and proportions for categorical variables and means (SD) or medians (first quartile, third quartile) for continuous variables. Differences in characteristics were tested using Fisher’s Exact tests for categorical variables and T-test or Wilcoxon rank-sum for continuous variables. Characteristics examined included baseline characteristics such as healer’s age, sex, education and marital status. Also included were, relationship status, years of practice and HIV test status at baseline.

At baseline and endline, the HIV transmission knowledge and correctness of techniques for PPE donning and doffing were separately assessed and compared between the study arms. Over the course of 6 months, on a monthly basis, the self-reported use of gloves was assessed and compared. SAS v9.4 (SAS Institute, Cary, NC) was used for all analyses and significance was set at α = 0.05.

### Ethical approvals

Ethical approval for this study was received from the Vanderbilt Institutional Review Board (IRB # 200298) and the University of Witwatersrand Human Research Ethics Committee (Medical) (IRB #M200229), as well as the Mpumalanga Provincial Department of Health’s Research Ethics Committee. All study participants provided written informed consent. The clinical trial was registered with clinicatrials.gov (NCT04440813).

## Results

One hundred and thirty-six traditional healers in Bushbuckridge, South Africa participated in this study to improve their use of PPE during patient encounters. Participants were largely female (80%), were an average of 51 years old, practiced as traditional healers for an average of 17 years, had achieved a primary school education (80%), and spoke xiTsonga (96%). Forty-three percent of healers were married or living with a partner and 49% either disclosed themselves as HIV+ or received a positive HIV test result at study baseline (Table 1).

**Table 1:** Participant demographics.

	Healer-trained (N = 67)	HCW trained (N = 69)	Total (N = 136)
**Healer’s Age**			
N	67	69	136
Median (IQR)	56.0 (42.0, 63.0)	51.0 (40.0, 57.0)	52.5 (40.5, 60.5)
**Healer’s Sex**, n (%)			
Male	14 (20.9%)	12 (17.4%)	26 (19.1%)
Female	53 (79.1%)	57 (82.6%)	110 (80.9%)
**Healer’s Education**, n (%)			
More than Primary education	13 (19.4%)	12 (17.4%)	25 (18.4%)
Primary education	54 (80.6%)	55 (79.7%)	109 (80.1%)
Missing	0 (0.0%)	2 (2.9%)	2 (1.5%)
**Languages Spoken**, n (%) (Totals >100% due to individuals speaking multiple languages)			
English, n (%)	6 (9.0%)	15 (21.7%)	21 (15.4%)
Shangaan, n (%)	65 (97.0%)	66 (95.7%)	131 (96.3%)
Afrikaans, n (%)	2 (3.0%)	0 (0.0%)	2 (1.5%)
Portuguese, n (%)	3 (4.5%)	1 (1.4%)	4 (2.9%)
Zulu, n (%)	20 (29.9%)	24 (34.8%)	44 (32.4%)
Xhosa, n (%)	6 (9.0%)	7 (10.1%)	13 (9.6%)
Other, n (%)	7 (10.4%)	15 (21.7%)	22 (16.2%)
**Number of languages spoken**			
N	67	69	136
Median (IQR)	1.0 (1.0, 2.0)	2.0 (1.0, 2.0)	1.0 (1.0, 2.0)
**Healer’s Marital Status**, n (%)			
Living with partner/Married	30 (44.8%)	28 (40.6%)	58 (42.7%)
Not living with Partner (single, widowed or divorced)	37 (55.2%)	40 (58.0%)	77 (56.6%)
Missing	0 (0.0%)	1 (1.4%)	1 (0.7%)
**How many years have you practiced as a traditional healer?**			
N	67	69	136
Mean (SD)	18.7 (14.20)	16.0 (13.87)	17.3 (14.05)
Median (IQR)	20.0 (6.0, 28.0)	10.0 (4.0, 25.0)	16.0 (5.0, 28.0)
**HIV status at baseline**, n (%)			
Positive	35 (52.2%)	32 (46.4%)	67 (49.3%)
Negative	26 (38.8)	32 (46.4%)	58 (42.6%)
Not tested	6 (9.0%)	5 (7.2%)	11 (8.1%)

At baseline prior to training, healers understood that re-using needles (95% answered correctly) and sharing blades during incisions (93% answered correctly) could transmit HIV. However, some healers (41%) believed that HIV was caused by a curse, or that a person can get HIV by sharing forks, spoons, or cups with a person who has HIV (23%). The only statistically significant difference in knowledge between the two groups was knowledge of HIV transmission from mosquito bites. Participants in the healer trained group were more likely to believe transmission was possible (66%) compared to healers in the HCW trained group (42%; p = 0.006) (See Table 2).

**Table 2:** Baseline knowledge about HIV transmission.

	Healer-trained (N = 67)	HCW trained (N = 69)	Total (N = 136)	P-value
**A person can get HIV by getting an injection with a needle that was already used on someone else.**, n (%)				0.674^2^
True	62 (92.5%)	67 (97.1%)	129 (94.8%)	
False/Unsure	3 (4.5%)	2 (2.9%)	5 (3.7%)	
Missing	2 (3.0%)	0 (0.0%)	2 (1.5%)	
**A person can get HIV by sharing blades.**, n (%)				0.487^2^
True	61 (91.0%)	66 (95.7%)	127 (93.4%)	
False/Unsure	5 (7.5%)	3 (4.3%)	8 (5.9%)	
Missing	1 (1.5%)	0 (0.0%)	1 (0.7%)	
**A person can get HIV from mosquito bites.**, n (%)				0.006^2^
True/unsure	44 (65.7%)	29 (42.0%)	73 (53.7%)	
False	22 (32.8%)	40 (58.0%)	62 (45.6%)	
Missing	1 (1.5%)	0 (0.0%)	1 (0.7%)	
**A person can get HIV by sharing forks, spoons or cups with a person who has HIV.**, n (%)				0.097^2^
True/unsure	19 (28.4%)	11 (15.9%)	30 (22.1%)	
False	47 (70.1%)	58 (84.1%)	105 (77.2%)	
Missing	1 (1.5%)	0 (0.0%)	1 (0.7%)	
**A person can get HIV from a curse.**, n (%)				1.000^2^
True/unsure	26 (38.8%)	27 (39.1%)	53 (39.0%)	
False	38 (56.7%)	42 (60.9%)	80 (58.8%)	
Missing	3 (4.5%)	0 (0.0%)	3 (2.2%)	
**Using gloves during “injections” will prevent HIV transmission.**, n (%)				0.719^2^
True	63 (94.0%)	64 (92.8%)	127 (93.4%)	
False/Unsure	3 (4.5%)	5 (7.2%)	8 (5.9%)	
Missing	1 (1.5%)	0 (0.0%)	1 (0.7%)	

^2^Fisher Exact test

Healers were tested in their ability to don, doff, and dispose of PPE immediately after the initial one-day training session and at 7 months after enrollment. Healers in the HCW arm showed equivalent PPE scores as those trained by traditional healers at 7-months (PPE total median donning and doffing skills completed correctly in health care worker trained 16.0 (IQR: 14, 20) vs. 16.6 (IQR: 14, 20) among those trained by healers). Key steps, including hand hygiene, donning, and doffing the glove correctly, were completed correctly by more than 80% of participants. We learned, during the study, that some healers in both arms were using PPE but were cutting the tip of the glove off on their dominant hand so they could maintain better control of the razor. This was addressed during in-person visits and at the end of the study 86% of healers recognized that this behavior increased their changes of contracting HIV.

We captured self-reported glove use during each treatment conducted with a razor. Healers in both arms self-reported high levels of glove use during”injections”, with no statistical difference by study arm (p = 0.934). When we assessed actual glove and razor blades disposed each month, a similar trend emerged (Table 3). Healers self-reported no re-use of gloves or razor blades.

**Table 3:** Number of gloves used per “injection” conducted (based on PPE collected).

		Healer Trained (n = 67)	Health Care Worker Trained (n-69)	Total (n = 136)	p-value
Month 1	N	27	39	66	0.941^1^
	Mean (SD)	1.2 (0.82)	1.2 (1.27)	1.2 (1.10)	
Month 2	N	37	45	82	0.763^1^
	Mean (SD)	2.0 (1.18)	2.0 (1.30)	2.0 (1.24)	
Month 3	N	34	38	72	0.538^1^
	Mean (SD)	2.0 (0.53)	2.1 (0.83)	2.1 (0.70)	
Month 4	N	21	37	58	0.425^1^
	Mean (SD)	2.4 (1.24)	2.1 (0.75)	2.2 (0.95)	
Month 5	N	20	29	49	0.311^1^
	Mean (SD)	2.3 (1.11)	2.1 (0.71)	2.2 (0.90)	
Month 6	N	21	33	54	0.731^1^
	Mean (SD)	2.1 (0.44)	2.1 (0.41)	2.1 (0.42)	
**Overall**	N	53	60	113	0.888^1^
	Mean (SD)	1.9 (0.73)	1.9 (0.79)	1.9 (0.76)	

^1^t-test

Healers in both the HCW- and healer-trained groups struggled in the first month after training to use gloves during each “injection” (anecdotally, many were using only a single glove to conserve materials), by the end of the study they were using 2 gloves for each “injection” 100% of the time. We offered HIV testing to those healers with a negative baseline test; none seroconverted during the study period (with one person refusing the re-test).

## Discussion

Trained “early adopter” traditional healers successfully delivered PPE training to 67 healer participants in a rural South African setting with a paucity of allopathic HCWs. The study findings indicate that there were no significant differences in the levels of PPE donning and doffing skills and rates of PPE use between healers trained by HCWs, and those trained by fellow healers themselves over a six-month period. There are an estimated 200,000 traditional healers in South Africa, [1–5] all of whom need access to PPE training and supplies to prevent blood borne infections, including HIV and Hepatitis B or C. This result suggests that training of correct PPE by other healers was as effective as the training provided by HCWs in terms of imparting necessary skills and knowledge for correct PPE usage. It highlights the potential and feasibility of peer led programs in achieving comparable outcomes to HCWs led training programs.

The lack of significant differences in PPE usage rates between the two groups implies that traditional healers can effectively disseminate PPE knowledge and skills to their peers. These findings are significant as it supports the idea of utilizing traditional healers as trainers, not only to enhance the adoption of PPE, but to overcome barriers such as limited access to healthcare facilities and cultural differences. These findings emphasize the potential of utilizing the existing network and expertise of traditional healers to scale up PPE training initiatives nationwide.

The need for PPE support among traditional healers cannot be ignored. With 44% of healers testing HIV+ in our sample, healers remain an unidentified high-risk group in the region. Other studies have shown higher than expected levels of HIV among traditional healers due, at least in part, to blood exposure during treatments [6, 39]. Healers have inconsistent access to training and gloves from the health system in South Africa [23] making it difficult for them to acquire and use PPE regularly and with fidelity.

Scaling up a PPE program will be facilitated by healers being extremely interested in engaging with the allopathic health system [23, 40–42]. Healers want to learn to deliver HIV and TB diagnostic, referral, and care services but PPE distribution will be necessary to facilitate this support [1, 43–47]. Despite the positive work healers can contribute, barriers to PPE distribution scale up will likely include financial resources, insufficient stock of PPE, and a mutual suspicion between healers and Department of Health staff. Until healers are convinced that their supply of PPE will continue without disruption, healers will continue to hoard PPE.

### Strengths and limitations

This study clearly demonstrated the traditional healers trained by other traditional healers on the donning, doffing and use of PPE performed no differently than healers trained by HCWs. This study was undertaken in a setting where an intervention like this is clearly relevant–with a prevalence of HIV among healers found to be 44%. In addition to protecting seronegative healers from contracting HIV from their clients, the use of PPE also protects healer’s clients from acquiring HIV from the healer. However, this study was not designed to examine this and while this argument further supports the use of PPE by traditional healers, further research can assist in determining the full benefit of PPE usage (to both the traditional healer and their client). Furthermore, this study was conducted in a rural context that is largely representative of much of rural South Africa, though the results may not be generalizable to more urban settings. Traditional healers may have varying levels of knowledge and skills themselves, which could impact the consistency and quality of the training they provide to their peers. Ensuring standardized training content can be challenging when relying on peer trainers. However, this could be minimized by regular assessment, continuous feedback mechanisms and ongoing support and mentorship. As noted in the methods, this intervention was undertaken in a setting and population that has been part of research activities for more than three decades and while this was a strength in the present study, this also highlights the essential importance of strong, mutually respectful relationships between researchers, healers and HCWs- and may be a limitation in other settings where such relationships do not exist. Lastly, we were unable to verify if healers used PPE or razor blades more than once. All data on this was self-report but we do not think they would have had any motivation to lie, given that PPE was given freely distributed and because it has become standard practice for patients to bring their own razor blade for treatments [23].

## Conclusion

Training and distribution of PPE, led by trained traditional healers in South Africa, is both a feasible and effective strategy to increase use of gloves during treatments that can result in blood exposures. While healers are not considered a high-risk population, with an HIV prevalence of 44%, this group needs to receive additional consideration and support to reduce their risk of HIV acquisition. Scaling up a PPE program will require collaborations between Department of Health, traditional healer association and other relevant key individuals and a train-the-trainer model to facilitate capacity building and education of healer leaders around the country. Together, they can develop culturally sensitive training programs, address logistical challenges, including the availability of PPE supplies and suitable infrastructure. With the estimated large number of traditional healers in South Africa, this approach can offer an inexpensive and efficient strategy to reach significant portion of the traditional healer community, ensuring widespread adoption of proper PPE practices and ultimately reducing the risk of infection transmission.

## Supporting information

S1 Checklist CONSORT checklist. (DOCX)
